# Omega Fatty Acids in Hair Loss Disorders: A Scoping Review of Preclinical and Clinical Evidence

**DOI:** 10.1111/jocd.71184

**Published:** 2026-09-07

**Authors:** Nana‐Hawwa Abdul‐Rahman, Jacqueline D. Wang, Miriam Motlak, Gerardo Guiltarte, Reethi S. Padmanabhan, Ellen T. Lee, Sonal Choudhary

**Affiliations:** ^1^ University of Pittsburgh School of Medicine Pittsburgh US; ^2^ Department of Dermatology University of Pittsburgh School of Medicine Pittsburgh US

**Keywords:** alopecia, dermal papilla cells, dietary supplements, hair follicle biology, hair growth, omega fatty acids, omega‐3, omega‐6, omega‐9

## Abstract

**Background:**

Omega fatty acids, including polyunsaturated omega‐3 and omega‐6 fatty acids (PUFAs) and monounsaturated omega‐9 fatty acids, regulate inflammation, cellular signaling, and tissue homeostasis, and may influence hair follicle biology and hair growth. The widespread use of dietary supplements for hair loss has been fueled by increasing consumer interest and the rapid spread of health information on social media.

**Aims:**

This scoping review synthesizes preclinical and clinical evidence on the role of omega fatty acids in the management of alopecia.

**Methods:**

PubMed, Embase, ClinicalTrials.gov, the Cochrane Library, and Ovid MEDLINE were searched from database inception through February 2, 2026. The review was conducted in accordance with the PRISMA‐ScR. Eligible studies were peer‐reviewed, English‐language studies evaluating the relationship between omega fatty acids and hair growth, hair loss, or hair follicle biology in scarring or nonscarring alopecia.

**Results:**

Preclinical studies suggest that omega fatty acids may promote dermal papilla cell proliferation, activate signaling pathways involved in hair follicle growth, suppress mediators of androgen‐driven hair loss, and may protect against chemotherapy‐induced follicular apoptosis. Clinical studies have reported improvements in hair density, hair growth, and reduced shedding, although many studies evaluated combination nutritional supplements rather than omega fatty acids alone. In contrast, one clinical study of men with androgenetic alopecia found that higher omega‐3 intake was associated with a lower likelihood of clinical improvement. Limited clinical evidence also suggests that omega fatty acid supplementation may support hair regrowth in alopecia areata through immunomodulatory and anti‐inflammatory effects.

**Conclusion:**

Current data do not support omega fatty acid supplementation as a standalone treatment for any alopecia subtype.

## 1 Introduction

Hair follicle cycling consists of four sequential phases: anagen (active growth), catagen (regression), telogen (resting), and exogen (shedding) [1, 2]. Normal scalp hair spends approximately 85%–90% of its time in the anagen phase [1]. Many alopecia disorders arise from disruptions in these tightly regulated transitions, leading to shortened anagen duration, premature catagen entry, prolonged telogen, or excessive hair shedding [1, 2]. Alopecia represents a group of hair loss disorders classified as non‐scarring (non‐cicatricial) or scarring (cicatricial) [3] In non‐scarring alopecia, hair follicles remain intact, allowing for potential regrowth [3]. Conversely, scarring alopecia results from chronic inflammation that irreversibly destroys hair follicular stem cells, causing permanent hair loss [3]. Beyond its clinical presentation, alopecia also has significant psychological effects and can reduce quality of life [4, 5, 6]. Treatment options for alopecia range from pharmacologic therapies to procedural interventions and are often associated with financial, accessibility, and tolerability challenges [7]. To circumvent these barriers, there is growing interest in affordable and noninvasive alternatives, including over‐the‐counter dietary supplements [2, 8, 9].

Among these, omega fatty acids (omega‐3, ‐6, and ‐9) have gained attention for their broad dermatologic benefits, including promoting hair growth [9, 10]. Overall, their anti‐inflammatory, immunomodulatory, and barrier‐stabilizing properties contribute to scalp health [11, 12, 13, 14, 15, 16]. Studies have suggested that they exert diverse biological effects depending on their molecular structure and the type of alopecia [9, 17]. For example, γ‐linolenic acid inhibits 5α‐reductase, reducing the conversion of testosterone to 5α‐dihydrotestosterone (5α‐DHT), a key driver of androgenetic alopecia [18, 19]. Interest in nutraceuticals has grown in recent years, fueled in part by social media and direct‐to‐consumer marketing [20, 21, 22, 23]. Coupled with the lack of FDA premarket review for dietary supplements, evidence‐based studies are crucial for dermatologists counseling patients seeking omega fatty acid supplementation for alopecia [24]. Similar enthusiasm previously surrounded biotin supplementation for hair loss, although subsequent evidence demonstrated benefit primarily in patients with true biotin deficiency [25, 26]. As omega fatty acid supplementation continues to gain popularity, a critical appraisal of the current preclinical and clinical evidence is needed. Therefore, this scoping review synthesizes the available evidence on the efficacy of omega fatty acids in the prevention and management of various types of alopecia.

## 2 Methods

Given the substantial heterogeneity in study designs, populations, and outcome measures across the preclinical and clinical literature on omega fatty acids and hair loss, a scoping review methodology was selected to map the breadth and nature of the available evidence rather than to pool effect estimates or formally rate certainty of evidence. This review followed the Joanna Briggs Institute methodology for scoping reviews and is reported in accordance with the PRISMA extension for Scoping Reviews (PRISMA‐ScR; a completed checklist is provided as Data S1). A comprehensive literature search was performed in PubMed, Embase, ClinicalTrials.gov, the Cochrane Library, and OVID/Medline from database inception to February 2, 2026. The search strategy combined controlled vocabulary and keywords related to omega fatty acids and hair loss disorders. See Data S2 for the search strategy, which was adapted for each database. Only peer‐reviewed studies with clearly defined methodologies were included in this review. Studies were included if they evaluated omega fatty acids in relation to hair growth, hair loss, or hair follicle biology. Both scarring and non‐scarring alopecia were considered. Eligible study designs included randomized controlled trials, cohort studies, case–control studies, cross‐sectional studies, case reports and case series, animal studies, and in vitro experiments involving hair follicles, dermal papilla cells, keratinocytes, or immune pathways relevant to hair biology. Studies were required to report outcomes related to hair growth, hair density, hair shedding, hair cycle dynamics, follicular inflammation, or fibrosis. Only English‐language peer‐reviewed articles were included. Studies were excluded if they lacked hair‐related outcomes, were not peer‐reviewed, or did not have full‐text availability. All identified records were imported into Rayyan and duplicates were removed. Two reviewers (NA and JW) independently screened titles and abstracts for eligibility. Full texts of potentially relevant articles were subsequently reviewed for inclusion. Disagreements during screening were resolved by MM. Data were extracted using a standardized data collection form. Extracted variables included study characteristics (author, year, country, and study design), alopecia subtype, population or experimental model, and type and formulation of omega fatty acid exposure. Findings were synthesized narratively, with clinical and preclinical evidence evaluated separately and interpreted according to study design and overall strength of evidence.

## 3 Results

A total of 1376 records were identified; 577 duplicates were removed. After title and abstract screening, 24 full‐text articles were assessed for eligibility, and 21 studies met the inclusion criteria. Studies involving any alopecia subtype were eligible; however, the available literature was largely limited to non‐scarring alopecia and experimental hair growth models. Of the included studies, 15 were preclinical studies (including in vitro, ex vivo, and animal studies) and 6 were clinical studies, including clinical trials, an observational cohort study, and case reports. Most studies evaluated omega fatty acids as components of multi‐nutrient formulations rather than as isolated interventions, which contributed to heterogeneity in the available evidence (Table 1).

**TABLE 1:** Summary of included studies.

References	Study type	Alopecia type	Intervention/product	Omega fatty acid type	Sample size *n*	Route of administration	Duration
Ahmed 2026	Preclinical (rats)	Unspecified hair growth	Topical Opophytum forskahlii Seed Oil (OFSO)	Oleic acid (omega‐9), Linoleic acid (omega‐6)	3 per group (control, OFSO, 2% MNX)	Topical	2 weeks
Dong 2025	Preclinical (mice)	Androgenetic alopecia	Topical conjugated linoleic acid–loaded nanovesicles (Arg‐CLAVs) and minoxidil‐loaded nanovesicles (MNX@Arg‐CLAVs)	Linoleic acid (omega‐6)	Male: 5 per group (Control, Model, 5% MNX, 0.45% MNX Arg‐CLAVs, MNX@Arg‐CLAVs); Female: 4 per group (Control, Model, 5% MNX, MNX@Arg‐CLAVs)	Topical	27 days (male model); 34 days (female model)
Ying 2024	Preclinical (in vivo and in vitro: HFDPCs and mice)	Unspecified hair growth	Nannochloropsis salina Fermented Oil (NSO)	Eicosapentaenoic acid (omega‐3), linoleic acid (omega‐6)	In vitro: 5 × 10 [3] cells/well (in triplicate); In vivo: 8 per group (Control, solvent, 3% MNX, 3% NSO, 5% NSO)	In vitro cell culture; Topical	In vitro: 60 h; In vivo:14 days
Shi 2024	Clinical (human participants)	Male pattern hair loss	Dietary intake of omega‐3 and ‐6	Omega‐3 and 6 fatty acids	15 813	Oral	Not specified
Lourith 2021	Preclinical (HFDPCs)	Unspecified hair growth	Para rubber seed oil	Oleic acid (omega‐9)	1 × 10 [4] cells/well (in triplicate)	In vitro cell culture	5 days
Ryu 2021	Preclinical (HFDPCs)	Unspecified hair growth	*Malva verticillata* Seed	Linoleic acid (omega‐6)	2 × 10 [4] cells/well (in triplicate)	In vitro cell culture	2 days
Kang 2018	Preclinical (mice)	Unspecified hair growth	Mackerel‐derived fermented fish oil (FFO) extract and its component docosahexaenoic acid (DHA)	omega‐3 fatty acid	8 per group (Control, FFO extract, 5% MNX)	Topical	5 weeks
Wainwright 2018	Clinical (human participants)	Unspecified hair growth	Supplement capsule	omega‐3 fatty acid	161	Oral	14 weeks
Hamel 2017	Preclinical (monkeys)	Unspecified Alopecia	Oral Omega‐3 chewable gummy supplement (Yummi BearsTM, Hero Nutritionals; Santa Ana, CA, USA)	omega‐3 fatty acid	6	Oral	5 months (Sept 2014‐Jan 2015)
Nichols 2017	Clinical (human participants)	Androgenetic alopecia	Cholecalciferol, omega‐3 and ‐6 fatty acids, melatonin, antioxidants, and botanical 5‐alpha reductase inhibitors	Omega‐3 and 6 fatty acids	10	Oral	24 weeks
Harvey 2020	Clinical (human participants)	Alopecia areata	Supplement	Omega‐3 fatty acids	1	Oral	5 months
Truong 2017	Preclinical (mice)	Androgenetic alopecia	Red Ginseng Oil (RGO)	Linoleic acid (omega‐6)	5 per group (Control, 0.5% TES, TES + 10% RGO, TES + 1% LA, TES + 1% SITOS, TES + 1% BICYCLO, TES + Finasteride)	Topical	4 weeks
Floc'h 2015	Clinical (human participants)	Female pattern hair loss	Oral essential fatty acid–rich nutritional supplement containing fish oil (omega‐3), blackcurrant seed oil (omega‐6, including GLA), lycopene, and vitamins C and E, once daily for 6 months.	Omega‐3 and ‐6 fatty acids	79 (Supplement), 39 (Control)	Oral	6 months
Choi 2014	Preclinical (mice)	Unspecified hair growth	Topical 3% rice bran supercritical CO2 extract (RB‐SCE)	Linoleic acid (omega‐6)	5 per group (Control, 3% MNX, RB‐SCE, Linoleic acid, policosanol, Oryzanol, 𝛾‐tocotrienol)	Topical	4 weeks
Munkhbayar 2016	Preclinical (in/ex vivo and in vitro: HFDPCs, hair follicle organ culture, and mice)	Unspecified hair growth	Topical and/or in vitro application of arachidonic acid (AA) to human dermal papilla cells and mouse skin	Arachidonic acid (omega‐6)	In vitro: 1 × 10^4^ cells/well; Ex vivo: 400 hair follicles; In vivo: 5 per group (Control, 3% MNX, 2% AA)	In vitro cell culture, Topical	In vitro: 1 day, Ex vivo: 12 days, In vivo: 4 weeks
Vingler 2007	Preclinical (human hair follicles)	Unspecified hair growth	6‐O‐GL, together with 3‐O‐GL	Oleic acid (omega‐9)	12 follicles per treatment condition (Control, varying doses of glucose, LA, 6‐O‐GL) in triplicate	In vitro culture	10 days
Takahata 1999	Preclinical (rats)	Chemotherapy‐Induced Alopecia	Docosahexaenoic acid (DHA) administered with chemotherapy (cyclophosphamide) in rats	Docosahexaenoic Acid (omega‐3)	Not specified	Oral	9 days
Munnich 1980	Clinical (human participants)	Alopecia totalis	Oral administration and cutaneous application of unsaturated fatty acid 2–400 mg, along with biotin supplementation	Oleic acid (omega‐9), linoleic acid (omega‐6), alpha‐linolenic acid (omega‐3), eicosatetraenoic acid (omega‐6)	1	Oral and topical	Up to 2 months
Hao 2022	Preclinical (mice)	Unspecified Alopecia	Fish‐oil high‐fat diet (omega‐3–enriched diet) in mice	Omega‐3 fatty acid	25 (low‐fat diet), 19 (fish oil high‐fat diet), 28 (cocoa butter high‐fat diet)	Oral	3 months
Skolnik 1977	Clinical (human participants)	Unspecified alopecia	Topical linoleic acid 150 mg/day (for 21 days) followed by continued use of safflower oil (containing 60%–70% linoleic acid).	Linoleic acid (omega‐6)	1	Topical	3 months

Abbreviations: 6‐O‐GL: 6‐O‐linoleyl‐D‐glucose; BICYCLO: bicyclo(10.1.0)tridec‐1‐ene; HFDPCs: Human follicular dermal papilla cells; LA: linoleic acid; MNX: minoxidil; SITOS: β‐sitosterol; TES: testosterone.

## 4 Discussion

### 4.1 Androgenetic Alopecia

#### 4.1.1 Evidence From Preclinical Studies

In androgenetic alopecia, hair cycle dynamics and changes in androgen signaling lead to follicle miniaturization and hair loss. Omega fatty acids have anti‐inflammatory and antioxidant properties, as evidenced by in vitro and in vivo preclinical studies. In a study of human follicle dermal papilla cells (HFDPCs), linoleic acid (omega‐6) was isolated from *Malva verticillata* seeds [19]. Treated HFDPCs exhibited Wnt/B‐catenin activation, increased papilla proliferation, and increased growth factors including VEGF, IGF‐1, and HGF [19]. Linoleic acid also inhibited DKK1, a key downstream mediator of androgen‐induced hair follicle regression [19]. This mechanistic work was conducted entirely in an in vitro HFDPC model without in vivo or clinical confirmation, so its translational relevance to human hair growth is yet to be established.

Beyond cultured cell models, a study using mice and dermal papilla cell cultures showed that conjugated linoleic nanovesicles reduced oxidative stress and the senescence‐associated secretory phenotype, while promoting dermal papilla cell proliferation and angiogenesis [27]. When combined with minoxidil, the nanovesicles improved hair regrowth in mice [27]. An additional preclinical study investigated the effects of daily red ginseng oil, which contains linoleic acid on mouse models of androgenetic alopecia for 28 days [28]. The authors observed increased hair regenerative capacity, increased activation of both Wnt/β‐catenin and Cyclin D1/Cyclin E, and increased expression of Bcl‐2 (an anti‐apoptotic protein) [28]. Another animal study supporting the role of omega fatty acids explored the benefits of arachidonic acid, an omega‐6 fatty acid involved in cell survival, angiogenesis, and follicular signaling [29]. Using human dermal papilla cells, hair follicle organ culture, and C57BL/6 mice, arachidonic acid administration increased growth factors FGF‐7 and FGF‐10, activated critical signaling pathways including ERK, CREB, and AKT signaling, and induced anagen phase in mice [29].

#### 4.1.2 Evidence From Clinical Studies

A randomized comparative clinical study evaluated whether nutritional supplementation with omega‐3 and omega‐6 fatty acids (plus antioxidants) could improve hair density and hair loss in 120 healthy women with Ludwig Stage 1 female pattern hair loss (FPHL) [17]. Baseline plasma fatty‐acid levels were not assessed or reported. However, participants with conditions that could contribute to hair loss or influence study outcomes, including iron, zinc, or vitamin B6 deficiencies, pregnant or postpartum were excluded. Thus, the study did not evaluate baseline nutritional status as a study outcome but attempted to minimize potential confounding from nutritional deficiencies through its exclusion criteria [17]. At 6 months, participants demonstrated increased hair density, reduced telogen hair percentage, and increased thicker anagen hairs (> 40 μm) [17].

An additional clinical study evaluated the effects of a daily oral supplement on 10 patients with androgenetic alopecia [30]. Participants received a supplement containing omega‐3 and omega‐6 fatty acids, along with melatonin, Vitamin D, soy isoflavones, and green tea extract [30]. After 24 weeks of daily treatment, 80% of subjects improved based on investigator assessment, with increased hair mass index and terminal hair counts [30]. This study was limited by its small sample size (*n* = 10), open‐label design without a placebo comparator, and reliance on investigator‐assessed improvement, each of which increases susceptibility to bias [30]. Similar to the prior study, the authors did not measure baseline plasma or serum fatty‐acid status, dietary essential fatty acid intake, or screened participants for an underlying nutritional deficiency before treatment [30]. Additionally, both studies evaluated omega fatty acids within multi‐ingredient formulations, which limits attribution of the observed benefit to omega fatty acids alone [17, 30].

Lastly, a population‐based prospective cohort study using participants from the UK Biobank examined individuals with male pattern hair loss (*n* = 15 813) [31]. Baseline and average dietary n‐3 and n‐6 fatty‐acid intakes were estimated using 24‐h dietary recalls and categorized into quartiles. The study therefore evaluated dietary fatty‐acid exposure but did not measure baseline plasma fatty‐acid concentrations. The analysis adjusted for demographic, behavioral, mental health, genetic, energy, and total fat intake variables; however, nutritional deficiencies were not specifically reported as assessed or adjusted for. Participants were grouped according to dietary recall, self‐reported visual assessments of hair loss, and average intake of omega‐3 and omega‐6 fatty acids [31]. Overall, 1022 men showed improvement in hair loss; however, participants with moderate omega‐3 intake were less likely to demonstrate improvement [31]. The inverse association observed in this cohort warrants further scrutiny, as it runs counter to much of the mechanistic and preclinical literature summarized above. Several explanations may account for this discrepancy. First, residual confounding and reverse causality are likely, as men with more visible or progressive hair loss may be more inclined to adopt omega‐3‐rich diets, which a cross‐sectional dietary assessment cannot exclude. Second, the study relied on self‐reported dietary recall and visually self‐assessed hair loss severity, both of which are prone to severe misclassification bias.

### 4.2 Chemotherapy‐Induced Alopecia, Alopecia Areata, Alopecia Totalis

#### 4.2.1 Evidence From Preclinical Studies

Chemotherapy‐induced alopecia results from the preferential destruction of rapidly proliferating matrix keratinocytes within anagen hair follicles by cytotoxic chemotherapy [32, 33]. This leads to follicular apoptosis, premature hair shaft fracture, and rapid hair shedding [32, 33]. For chemotherapy‐induced alopecia (CIA), current evidence is primarily derived from preclinical studies. In a neonatal rat model, all animals treated with cytosine arabinoside or etoposide developed complete CIA, with apoptotic cells observed in skin sections [34]. Notably, rats receiving oral docosahexaenoic acid (DHA) were protected from CIA [34]. Although interleukin‐1α and lipopolysaccharide also conferred protective effects, the benefit of DHA was not inhibited by an interleukin‐1 receptor antagonist [34]. This suggests that DHA may act by modulating apoptosis rather than through the IL‐1 pathway [34]. Since CIA is believed to result from chemotherapy‐induced apoptosis in actively cycling follicular keratinocytes, DHA may protect hair follicles by reducing apoptosis [34]. This finding is derived entirely from a single animal model, and no human trial has tested whether the same protective effect translates to patients undergoing chemotherapy.

#### 4.2.2 Evidence From Clinical Studies

For alopecia areata, no preclinical studies were identified and only one clinical report described an 8‐year‐old boy with severe patchy alopecia areata affecting the scalp, eyebrows, and eyelashes [35]. He was managed with dietary counseling along with vitamin A 400 μg, vitamin D₃ 22.5 μg, zinc 10 mg, and 683 mg of total omega‐3 fatty acids [35]. Visible hair regrowth was observed by 2 months, and by 5 months the scalp had reportedly fully recovered, with regrowth of the eyebrows and eyelashes [35]. The omega‐3 component may have contributed to hair regrowth by dampening the autoimmune attack on hair follicles and promoting a more tolerogenic immune environment [35]. Alopecia areata is driven by cytotoxic CD8^+^ T cells and a Th1/Th17‐skewed response following collapse of hair follicle immune privilege [36]. Omega‐3 PUFAs (EPA/DHA) and their specialized mediators (e.g., resolvins, protectins, and maresins) suppress Th1/Th17 activity and CD8^+^/NK cell–mediated follicular attack [11, 15, 16]. They also expand FoxP3^+^ regulatory T cells, enhance tolerance to follicular antigens, and provide antioxidant effects [11, 15, 16]. Together, these mechanisms may reduce perifollicular inflammation and support reestablishment of hair follicle immune privilege in alopecia areata.

For alopecia totalis, Munnich et al. described a 12‐month‐old boy with multiple carboxylase deficiency who presented with alopecia totalis and dermatitis [37]. Despite a normal diet, the patient had low plasma linoleic acid (17.3% of total fatty acids compared with the expected 30%–35%) [37]. Oral and topical unsaturated fatty acid supplementation (200–400 mg/day orally with twice‐daily topical application), composed primarily of linoleic acid (C18:2, 71%), with oleic acid (C18:1, 11%), α‐linolenic acid (C18:3, 8%), and arachidonic acid (C20:4, 0.3%), led to rapid initiation of scalp hair growth and normalization of plasma linoleic acid levels (22.1%) [37]. Subsequent biotin therapy (10 mg/day) led to complete clinical and biochemical recovery [37]. Based on the clinical response, the authors concluded that linoleic acid deficiency contributed to the alopecia [37]. Omega fatty acid supplementation may have contributed to hair regrowth by restoring essential fatty acids critical for scalp and hair follicle health [37]. Ceramides, which are synthesized from linoleic acid in both the epidermis and hair follicles, are essential for barrier function and follicular integrity [38, 39, 40, 41]. Deficiencies can lead to scaling dermatitis, weakened hair shafts, and increased shedding [10, 40, 42, 43]. By replenishing essential fatty acids, omega supplementation may help restore ceramides, which are thought to support hair follicle health, encourage dermal papilla cell growth, and regulate important pathways like ERK and BMP/Wnt that control hair growth cycles [44, 45].

### 4.3 Unspecified Alopecia

#### 4.3.1 Evidence From Preclinical Studies

Kang et al. examined mackerel‐derived fermented fish oil (FFO) containing DHA and EPA [45]. In ex vivo follicle cultures, FFO increased hair fiber length compared to minoxidil and controls [45]. Subsequent experiments showed that FFO treatment in cultured dermal papilla cells increased cell proliferation and cell cycle progression compared to controls [45]. Mechanistically, FFO activated extracellular signal‐regulated kinase (ERK), p38, and Akt signaling pathways, which are associated with cell proliferation [45]. FFO extract was also found to promote nuclear translocation of β‐catenin through inactivation of glycogen synthase kinase‐3β (GSK‐3β) [45]. This suggests that FFO activated signaling pathways associated with dermal papilla proliferation and hair follicle growth [45].

Ying et al. also investigated a marine‐derived lipid product, Nannochloropsis salina fermented oil (NSO), which contains EPA along with other fatty acids [46]. In human dermal papilla cells, treatment with NSO increased cell proliferation and improved cell survival under oxidative stress comparable to, or in some conditions exceeding, that of minoxidil [46]. Gene expression analysis revealed upregulation of hair growth‐associated genes (including FGF2, MAPK3, and AKT2), and antioxidant genes (including TXNRD2, HMOX1, and GPX4) [46]. In mice, topical NSO increased hair length, hair weight, follicle number, and follicle depth, suggesting enhanced follicular growth [46].

Hamel et al. conducted an experimental study where six monkeys with mild alopecia received daily omega‐3 chewable gummies [47]. Alopecia scores decreased during treatment and slightly increased after treatment cessation [47]. Monkeys also exhibited reduced self‐grooming behaviors during treatment, which the authors suggested could indicate underlying subclinical inflammation [47]. This study involved a small number of animals (*n* = 6) without a randomized placebo‐controlled comparison group, and the concurrent reduction in self‐grooming behavior is itself a potential confounder for the alopecia outcome, complicating causal inference.

On the other hand, overconsumption of omega fatty acids may lead to opposing effects. In a study by Hao et al., mice fed a high‐fat diet rich in fish oil or cocoa butter developed severe non‐scarring patches of alopecia [48]. The authors found that this effect was linked to inflammatory macrophage accumulation around hair follicles [48]. These macrophages activated the TNF‐α and omega‐3 fatty acid/reactive oxygen species/IL‐36 signaling pathways [48]. However, this finding warrants cautious interpretation, as the study used group‐housed C57BL/6 mice, a strain well recognized for barbering and over‐grooming behaviors that can independently produce focal, non‐scarring alopecia and secondary perifollicular inflammation in group‐caged animals. Since barbering‐related hair loss and its associated inflammatory infiltrate can closely mimic a diet‐induced alopecia phenotype, housing‐related behavioral confounding cannot be excluded as a contributor to the findings, and the reported macrophage‐driven inflammatory mechanism may reflect, at least in part, a strain‐ and housing‐specific phenomenon rather than a generalizable effect of dietary omega‐3 fatty acid excess.

An in vivo study involving C57BL/6 mice demonstrated that topical application of a rice bran extract containing linoleic acid and γ‐oryzanol increased the formation of hair follicles histologically and promoted the expression of hair growth factors [49]. The in vitro study by Vingler et al. revealed that 6‐O‐GL, a glucose linoleate derivative promoted the elongation of human hair follicles in a dose‐dependent fashion [50]. Similarly, Munkhbayar et al. investigated the effects of arachidonic acid on hair growth in experimental models [29]. They found that human hair follicles treated with arachidonic acid for 12 days demonstrated significantly greater hair shaft elongation compared to vehicle‐treated controls [29]. The authors highlighted that arachidonic acid enhances hair growth in C57BL/6 mice and increases the duration of the anagen hair cycle [29].

Plant‐derived oils rich in omega‐6 fatty acids also promote hair growth. Ahmed et al. evaluated *Opuntia ficus‐indica* seed oil (OFSO), which is rich in linoleic acid [51]. In cultured human dermal fibroblasts, OFSO treatment resulted in a dose‐dependent increase in fibroblast proliferation while decreasing protein concentrations of collagenase, hyaluronidase, elastase, and tyrosinase [51]. Additionally, OFSO exhibited dose‐dependent suppression of COX1 and COX2 activity, suggesting potential anti‐inflammatory effects that may support skin and hair follicle health [51]. In rat models, topical OFSO treatment accelerated hair growth and increased the number of follicles in the anagen phase [51]. Network pharmacology analysis suggested that the oil may act through genes including PPARG, ESR1, and IL6, implicating pathways related to inflammation, lipid metabolism, and tissue regeneration [51].

Similarly, Lourith et al. investigated para rubber seed oil, which contains multiple fatty acids, including linoleic acid [18]. In cultured human dermal papilla cells, treatment with para rubber seed oil stimulated dermal papilla cell proliferation and showed antioxidant activity against oxidative stress [18]. Additionally, the oil had moderate inhibitory activity against 5α‐reductase in DU‐145 prostate carcinoma cells, but to a lesser extent compared to minoxidil and dutasteride treatments [18]. This suggests a possible mechanism for reducing androgen‐related hair loss.

#### 4.3.2 Evidence From Clinical Studies

Wainwright et al. examined an oral supplement containing omega‐3 fatty acids derived from fish oil combined with soy extract‐derived isoflavones, lycopene from tomato extract, and vitamins C and E [52]. In a quantitative survey‐based study involving 161 female participants consuming the supplement (three gel capsules daily) for 14 weeks, participants reported an improved perception of overall hair condition at 4 weeks, with continued perceived improvements at 9 and 14 weeks [52]. The area of greatest perceived improvement was reported to be hair growth, followed by hair strength and moisturization [52]. This was a survey‐based study relying on participants' subjective perception of hair improvement rather than objective measurement (e.g., trichoscopy, hair counts, or hair pull testing) and lacked a placebo comparator, limiting the strength of the evidence.

Skolnik et al. reported a case of a 19‐year‐old man who developed essential fatty acid (EFA) deficiency after 4 months of fat‐free intravenous hyperalimentation [53]. The patient presented with scalp dermatitis, diffuse scalp and eyebrow alopecia, and hair depigmentation [53]. Treatment involved daily topical linoleic acid (150 mg/day) applied for 21 days, followed by continued therapy with safflower oil containing 60%–70% linoleic acid [53]. Cutaneous scaling resolved within 3 weeks, while scalp and eyebrow hair regrowth and re‐pigmentation occurred after approximately 3 months of topical therapy [53].

### 4.4 PUFA‐Derived Metabolites and Hair Follicle Biology

Beyond their structural roles as membrane phospholipids, omega‐3 and omega‐6 polyunsaturated fatty acids can be converted into bioactive lipid mediators that may influence the hair‐growth cycle. For example, arachidonic acid, an omega‐6 fatty acid, is converted into prostaglandins such as PGE2 and PGD2 [54]. These prostaglandins appear to have different effects on hair growth. In men with androgenetic alopecia, PGD2 and its synthesizing enzyme, PTGDS, are increased in bald scalp compared with hair‐bearing scalp [55]. Experimental studies have also shown that PGD2 can inhibit hair‐follicle growth through GPR44/DP2 signaling [55]. In contrast, PGE2 may promote early hair‐follicle development and increase populations of hair‐follicle stem cells in preclinical models [56]. Therefore, simply measuring total omega fatty acid intake may not accurately predict its effects on hair growth. The outcome may depend on how much precursor is available, which enzymes and receptors are active, and whether more PGD2 or PGE2 is produced. This bidirectional effect on hair‐follicle cycle may provide an explanation for some of the observed heterogeneity in outcomes across studies.

Omega‐3 fatty acids, particularly EPA and DHA, can also be converted into specialized pro‐resolving mediators (SPMs), including resolvins, protectins, and maresins [57]. In experimental studies, some of these molecules reduce inflammatory signaling from immune cells, including CD8+, Th1, and Th17 cells, and may promote regulatory T‐cell activity [58, 59]. This is potentially relevant to alopecia areata, where immune cells attack the hair follicle after loss of its normal immune protection [60]. However, whether omega‐3–derived SPMs actually reduce inflammation or restore hair‐follicle immune privilege in patients with alopecia areata remains unproven.

Future studies should therefore look beyond omega fatty acid supplements. Measuring specific lipid mediators, including prostaglandins, leukotrienes, and SPMs, alongside clinical hair‐growth outcomes could help clarify how omega‐3 and omega‐6 fatty acids influence different hair‐loss disorders.

### 4.5 Mechanistic Heterogeneity: A Possible Role for the Gut Microbiota

The divergent effects of omega fatty acids observed across the reviewed preclinical and clinical studies, including the alopecia phenotype reported with high‐fat fish oil feeding and the inverse association observed in the UK Biobank cohort, may also be partly explained by inter‐individual and interspecies differences in gut microbial composition. Emerging evidence indicates that the gut microbiota shapes how dietary saturated and polyunsaturated fatty acids affect skin and hair follicle homeostasis [61]. This suggests that the same fatty acid exposure can yield divergent cutaneous and follicular effects depending on the host's underlying microbial community. Since none of the included preclinical or clinical studies characterized gut microbial composition alongside omega fatty acid exposure, this represents an unmeasured source of heterogeneity that could contribute to the discordant findings across animal models and human cohorts, and it may be a valuable variable for future studies to incorporate.

### 4.6 Strength and Limitations

The absence of confirmatory clinical trials does not establish a lack of biological activity; rather, it leaves open a modest supportive role for omega fatty acids in selected forms of alopecia, particularly as adjunctive rather than primary therapy.

A major limitation of the current literature is the paucity of well‐designed, controlled clinical trials. Within the available literature, many studies are limited by small sample sizes, lack of control groups, or retrospective design. Importantly, many clinical studies evaluated omega fatty acids in combination with other bioactive supplements (e.g., botanical extracts, antioxidants), making it difficult to isolate the independent effect of omega fatty acids. Preclinical and mechanistic studies provide important biological context, but their findings may not directly translate to clinical efficacy in humans.

## 5 Conclusion

Current evidence provides biologic plausibility that omega fatty acids influence hair follicle biology and may serve as adjunctive therapies for certain forms of alopecia. However, the available clinical evidence remains sparse, heterogeneous, and largely limited to multi‐nutrient supplementation studies, isolated case reports, or observational associations. Consequently, no conclusions regarding the independent clinical efficacy of omega fatty acids can presently be drawn. Existing evidence is strongest for mechanistic and preclinical effects in androgenetic alopecia, whereas evidence supporting chemotherapy‐induced alopecia, alopecia areata, alopecia totalis, and other alopecia subtypes remains insufficient. Large randomized trials evaluating isolated omega fatty acid supplementation are needed before routine clinical use can be recommended.

## Funding

The authors have nothing to report.

## Ethics Statement

The authors have nothing to report.

## Consent

The authors have nothing to report.

## Conflicts of Interest

The authors declare no conflicts of interest.

## Supporting information


**Data S1:** PRISMA‐ScR Checklist. Completed Preferred Reporting Items for Systematic Reviews and Meta‐Analyses Extension for Scoping Reviews (PRISMA‐ScR) checklist for the present scoping review.

**Data S2:** Search Strategy. Database‐specific search strategies combining controlled vocabulary and keywords related to omega fatty acids and hair loss disorders, adapted as appropriate for each database.

## Data Availability

Data sharing is not applicable to this article as no datasets were generated or analyzed during the current study.
