# Supplementary material for: Omega Fatty Acids in Hair Loss Disorders: A Scoping Review of Preclinical and Clinical Evidence
Source: J Cosmet Dermatol. 2026 Sep 7;25(9):e71184. doi: 10.1111/jocd.71184 (PMC13550812; doi:10.1111/jocd.71184)
Supplement: Supplementary file 2 [file JOCD-25-e71184-s002.docx]

**PubMed: 2/2/26**

**373 results**

(( "Fatty Acids"[MeSH] OR "omega fatty acid*"[tiab] OR omega-3[tiab] OR omega-6[tiab] OR omega-9[tiab] OR PUFA*[tiab] OR MUFA*[tiab] OR "eicosapentaenoic acid"[tiab] OR EPA[tiab] OR "docosahexaenoic acid"[tiab] OR DHA[tiab] OR "alpha-linolenic acid"[tiab] OR "linoleic acid"[tiab] OR "oleic acid"[tiab] OR "fish oil"[tiab] ) AND ( "Alopecia"[MeSH] OR "hair loss"[tiab] OR "hair follicle"[tiab] OR "non-scarring alopecia"[tiab] OR "telogen effluvium"[tiab] OR "androgenetic alopecia"[tiab] OR "female pattern hair loss"[tiab] OR "alopecia areata"[tiab] OR "scarring alopecia"[tiab] OR "cicatricial alopecia"[tiab] OR "lichen planopilaris"[tiab] OR "frontal fibrosing alopecia"[tiab] OR "central centrifugal cicatricial alopecia"[tiab] OR "folliculitis decalvans"[tiab] OR "discoid lupus erythematosus"[tiab]))

**Embase 860: 2/2/26**

| No. | Query | Results |
| --- | --- | --- |
| #31 | #23 AND #30 | 860 |
| #30 | #1 OR #3 OR #4 OR #5 OR #6 OR #25 OR #26 OR #28 OR #29 | 480847 |
| #29 | 'fatty acid' | 444425 |
| #28 | 'oleic acid' | 44209 |
| #27 | pufa | 21558 |
| #26 | 'omega 9 fatty acid' | 200 |
| #25 | 'omega 3 fatty acid' | 47166 |
| #23 | #2 OR #8 OR #9 OR #10 OR #11 OR #12 OR #13 OR #14 OR #15 OR #16 OR #17 OR #18 OR #19 OR #20 OR #21 OR #22 | 116566 |
| #22 | hair AND follicle | 25498 |
| #21 | discoid AND lupus AND erythematosus | 6511 |
| #20 | folliculitis AND decalvans | 539 |
| #19 | central AND centrifugal AND cicatricial AND alopecia | 452 |
| #18 | frontal AND fibrosing AND alopecia | 1326 |
| #17 | lichen AND planopilaris | 1383 |
| #16 | cicatricial AND alopecia | 1781 |
| #15 | 'scarring alopecia' | 2176 |
| #14 | 'alopecia areata' | 12194 |
| #13 | 'female pattern hair loss' | 834 |
| #12 | 'androgenetic alopecia' | 6284 |
| #11 | 'telogen effluvium' | 1243 |
| #10 | 'non scarring alopecia' | 474 |
| #9 | 'hair follicle' | 24423 |
| #8 | 'hair loss' | 26079 |
| #6 | 'fish oil' | 25102 |
| #5 | eicosapentaenoic AND acid | 17315 |
| #4 | 'alpha linolenic' AND acid | 2914 |
| #3 | 'omega 6 fatty acid'/exp OR 'omega 6 fatty acid' | 9582 |
| #2 | alopecia | 77410 |
| #1 | 'omega fatty acid' OR (('omega'/exp OR omega) AND fatty AND ('acid'/exp OR acid)) | 54909 |

**ClinicalTrials.gov: 32**

("fatty acid" OR "omega fatty acid" OR omega-3 OR omega-6 OR omega-9 OR PUFA OR MUFA OR "eicosapentaenoic acid" OR EPA OR "docosahexaenoic acid" OR DHA OR "alpha-linolenic acid" OR "linoleic acid" OR "oleic acid" OR "fish oil") AND (alopecia OR "hair loss" OR "hair follicle" OR "telogen effluvium" OR "androgenetic alopecia" OR "female pattern hair loss" OR "alopecia areata" OR "scarring alopecia" OR "cicatricial alopecia" OR "lichen planopilaris" OR "frontal fibrosing alopecia" OR "central centrifugal cicatricial alopecia" OR "folliculitis decalvans" OR "discoid lupus erythematosus")

**Cochrane Library: 24**

(( "Fatty Acids"[MeSH] OR "omega fatty acid*"[tiab] OR omega-3[tiab] OR omega-6[tiab] OR omega-9[tiab] OR PUFA*[tiab] OR MUFA*[tiab] OR "eicosapentaenoic acid"[tiab] OR EPA[tiab] OR "docosahexaenoic acid"[tiab] OR DHA[tiab] OR "alpha-linolenic acid"[tiab] OR "linoleic acid"[tiab] OR "oleic acid"[tiab] OR "fish oil"[tiab] ) AND ( "Alopecia"[MeSH] OR "hair loss"[tiab] OR "hair follicle"[tiab] OR "non-scarring alopecia"[tiab] OR "telogen effluvium"[tiab] OR "androgenetic alopecia"[tiab] OR "female pattern hair loss"[tiab] OR "alopecia areata"[tiab] OR "scarring alopecia"[tiab] OR "cicatricial alopecia"[tiab] OR "lichen planopilaris"[tiab] OR "frontal fibrosing alopecia"[tiab] OR "central centrifugal cicatricial alopecia"[tiab] OR "folliculitis decalvans"[tiab] OR "discoid lupus erythematosus"[tiab] OR "hair follicle*"))

**OVID/Medline: 87**

1	(omega fatty acid* or omega-3 or omega-6 or omega-9 or PUFA* or MUFA* or eicosapentaenoic acid or EPA or docosahexaenoic acid or DHA or alpha-linolenic acid or linoleic acid or oleic acid or fish oil).mp.	1242012

(Alopecia or hair loss or hair follicle* or non-scarring alopecia or telogen effluvium or androgenetic alopecia or female pattern hair loss or alopecia areata or scarring alopecia or cicatricial alopecia or lichen planopilaris or frontal fibrosing alopecia or central centrifugal cicatricial alopecia or folliculitis decalvans or discoid lupus erythematosus).mp.	498883

1 and 2

87
